# PAST AS PRESENT: HOW LIVED EXPERIENCE INFLUENCES INTERACTION WITH LTSS AMONG OLDER SPANISH SPEAKERS

**DOI:** 10.1093/geroni/igac059.1323

**Published:** 2022-12-20

**Authors:** Allen Glicksman, Misha Rodriguez, Lauren Ring, Michael Liebman

**Affiliations:** NewCourtland, Haverford, Pennsylvania, United States; APM, Philadelphia, Pennsylvania, United States; Private, Philadelphia, Pennsylvania, United States; IPQ Analytics Inc, Kennett Square, Pennsylvania, United States

## Abstract

Older persons who migrated to the mainland United States bring with them their experiences and culture from their homelands and the process of migration and settlement in their new communities. The attitudes, values and assumptions that emerge will contribute to the interaction of the individual with the formal Long Term Care Services and Support (LTSS) system. Our research focused on better understanding of that process among older Spanish speakers. Data from four focus groups (# of participants) conducted in Spanish about the participants experience with LTSS was used in this analysis. Findings demonstrate the importance of understanding the ways in which the worldview of these persons shape their expectations and use of the formal LTSS system. The findings will facilitate the development of a Social Interaction Model to enable planners, policy makers and practitioners in LTSS to more effectively work with older migrants.

